# Development of a multi-species luciferase-based double-antigen ELISA for the detection of antibodies against influenza A virus H5 clade 2.3.4.4b

**DOI:** 10.1128/jcm.01907-25

**Published:** 2026-08-13

**Authors:** Andrea Aebischer, Anne Günther, Ronja Piesche, Kerstin Wernike, Magdalena Murr, Timm Harder, Donata Hoffmann, Christian Grund, Beatriz Bellido Martín, Ron Fouchier, Katharina Daniel, Leon Ullrich, Rosanne Sprute, Christoph Kreer, Florian Klein, Marco Roller, Lukas Reese, Josefine Wassermann, Gereon Schares, Maryna Kuryshko, Elsayed M. Abdelwhab, Martin Beer

**Affiliations:** 1 Institute of Diagnostic Virology, Friedrich-Loeffler-Institut, Federal Research Institute for Animal Health, Greifswald, Germany; 2 Institute of Molecular Virology and Cell Biology, Friedrich-Loeffler-Institut, Federal Research Institute for Animal Health 39023, Greifswald, Germany; 3 Department of Viroscience, Erasmus Medical Center 6993, Rotterdam, the Netherlands; 4 Laboratory of Experimental Immunology, Institute of Virology, Faculty of Medicine and University Hospital Cologne, University of Cologne 686814, Cologne, Germany; 5 Cologne Excellence Cluster on Cellular Stress Responses in Aging-Associated Diseases (CECAD), Faculty of Medicine, Institute of Translational Research, University of Cologne 61059, Cologne, Germany; 6 German Center for Infection Research (DZIF), Partner Site Bonn-Cologne, Cologne, Germany; 7 Laboratory for Immunodynamics and Molecular Evolution, Institute of Virology, Faculty of Medicine and University Hospital Cologne, University of Cologne 61059, Cologne, Germany; 8 Zoological Garden Karlsruhe, Karlsruhe, Germany; 9 Institute of Epidemiology, Friedrich-Loeffler-Institut, Federal Research Institute for Animal Health 39023, Greifswald, Germany; University of California Davis, Davis, California, USA

**Keywords:** highly pathogenic avian influenza virus, HPAIV, H5N1, H5 clade 2.3.4.4b, serology, panzootic, antibodies, ELISA, surveillance, wildlife, birds, mammals, nanoluciferase

## Abstract

The highly pathogenic avian influenza viruses of subtype H5N1 represent a major threat to animal and public health. The current panzootic with H5 clade 2.3.4.4b has caused numerous, widespread outbreaks in various domestic and wild avian species with high mortalities, massive losses, and a high frequency of spillover events to unexpected novel mammalian hosts, such as dairy cows. The global H5N1 situation raises serious concerns about zoonotic risks due to effective mammal-to-mammal transmission. Therefore, it is critical to increase surveillance intensity of a broadened species range, particularly at the human-animal interface. For this purpose, reliable and cost-effective serological tools that are easy to perform and suitable for high-throughput screening are critically needed. The newly developed double-antigen enzyme-linked immunosorbent assay format employing a luminescence-based detection technology has demonstrated compliance with such prerequisites. The assay allowed sensitive and specific detection of antibodies directed against H5 hemagglutinin of clade 2.3.4.4b in a wide range of birds and mammals, including humans. Furthermore, it allowed differentiating H5 anti-head-specific from cross-reacting anti-stalk antibodies, which represents a valuable feature with regard to the monitoring of future vaccination programs with H5-specific vaccines. Thus, the assay is a significant contribution to existing serological diagnostic tests for a clade-optimized and species-independent detection of influenza A virus antibodies.

**IMPORTANCE:**

The ongoing highly pathogenic avian influenza virus H5N1 panzootic has caused numerous outbreaks in domestic and wild animals, with frequent spillover events to unexpected host species, which underscores the importance of intensified surveillance. However, sensitive and specific multi-species serological assays represent a major gap. For this purpose, we developed a novel double-antigen enzyme-linked immunosorbent assay that employs an innovative luminescence-based readout strategy. The test allowed a highly sensitive and specific detection of H5-specific antibodies in a wide range of avian and mammalian species, including humans. It therefore represents a valuable contribution to improving species-independent serological diagnostic tools for the detection of influenza A virus antibodies.

## INTRODUCTION

Infections with highly pathogenic avian influenza (HPAI) viruses have severe consequences for animal and human health, as currently demonstrated by the global spread of the HPAI H5N1 clade 2.3.4.4b. Since 2016, this clade caused outbreaks with devastating economic losses in domestic poultry, as well as previously unseen massive die-offs in different wild bird species (1). Furthermore, frequent spillovers in more than 80 mammalian species have been reported and are associated with high morbidities and mortalities (2–4), which raises serious concerns about the threat to endangered species and biodiversity (5, 6). The incursion of H5N1 into North and South America not only led to high numbers of fatalities among marine mammals but also revealed evidence of direct transmission between the animals (7–10). Mammalian transmission was previously also suspected during outbreaks in fur farms in Spain and Finland (11, 12). In 2024, H5N1 clade 2.3.4.4b was detected for the first time in dairy cattle in the United States and spread rapidly to affect more than 1,000 dairy farms in 18 U.S. states since that time (13). Subsequently, deadly infections of cats and other mammals in contact with highly infectious raw milk from affected farms, as well as of farm workers in close contact with infected poultry or cattle, were reported (14, 15). To date, sustained person-to-person transmission has not been documented, but the evidence of mammalian adaptations in the viruses isolated from different species proved the propensity of the virus to evolve toward more efficient replication in mammals, including humans (2, 16). All these studies emphasize the necessity of increasing surveillance intensity of a broadened spectrum of potential host species, especially at the human-animal interface. For this purpose, reliable multi-species serological tools are urgently needed (17). Enzyme-linked immunosorbent assays (ELISA) represent one of the most appropriate test systems since they are easy to conduct and suitable for automation, allowing a rapid and cost-effective analysis of large numbers of samples.

Over the last few years, several ELISAs for the detection of antibodies against hemagglutinin (HA) subtype H5 have been developed using different approaches. Even though antibody-competition formats allow a species-independent application, most assays have primarily been developed and validated for galliform poultry. Their performance in other domestic and wild avian species often showed diverging results, and comparative data for mammalian species are mostly not available (18–24). Though innovative, other detection strategies still rely on species- or antibody isotype-dependent reagents (25–29).

In order to enable a clade-optimized, specific detection of anti-H5 antibodies, which is completely independent of species and isotype, we developed a novel assay based on the previously described luciferase immunoprecipitation system (LIPS) technology (30, 31). The principle of the liquid-phase double-antigen ELISA is based on the capture of antibodies by their simultaneous binding to an immobilized capture antigen and a nanoluciferase-labeled detector antigen. The reporter gene produces a high-intensity, glow-type luminescence, which allows quantitation of antibody titers in small sample volumes with low background auto-luminescence (32). In addition, the system does not require purification of the detector antigen, which facilitates production and reduces costs. Our data show that the novel assay combines the outstanding sensitivity of the LIPS technique with a fast turnaround time and the capability for robust high-throughput application of a standard solid-phase ELISA. Thus, the format represents a valuable and versatile contribution to existing H5-specific serological tests to improve antibody detection in a broad range of host species.

## MATERIALS AND METHODS

### Cloning

The full-length H5 HA protein, comprising amino acids (aa) 17 –530 from influenza virus isolate A/arctic tern/Germany-SH/AI03798/2022 (A/H5N1, clade 2.3.4.4b) (EPI2242291), was amplified from a codon-optimized synthetic gene (GeneArt, Regensburg, Germany) and cloned into the expression vector pEXPR103 (iba lifesciences, Göttingen, Germany) in frame with an N-terminal modified mouse Igκ light chain signal peptide. C-terminally, a T4 fibritin foldon trimerization domain (GYIPEAPRDGQAYVRKDGEWVLLSTFL) and a FLAG-tag were added. The original H5 sequence was additionally modified by introducing the aa exchange R345Q to prevent cleavage of the full-length H5. To obtain a soluble HA1 subdomain (aa 17 –345), aa 346 –530 and the T4 fibritin sequence were deleted from the full-length construct using a restriction-free PCR cloning technique (33). Primer sequences are available upon request.

To produce the H5-nanoluciferase antigen (H5-Nano), the nanoluciferase reporter gene (AFI79290.1) was synthetically produced (GeneArt, Regensburg, Germany) and added C-terminally to the full-length H5 construct. The T4 fibritin sequence was removed, and a 12-aa linker sequence (GGSQSDSRGGNG) was inserted upstream of the C-terminal FLAG-tag.

### Protein expression

The recombinant full-length H5 fused to nanoluciferase (H5-Nano), the full-length trimeric H5, and the HA1 subdomain were expressed in Expi293 cells. The cells were grown in Expi293 expression medium (Thermo Fisher Scientific, Waltham, Massachusetts, USA) and polycarbonate Erlenmeyer flasks (Corning, New York, USA) at 37°C, 8% CO_2_, 125 rpm. Transfection was performed using the ExpiFectamine293 transfection kit (Thermo Fisher Scientific) according to the manufacturer’s instructions, and the cell culture supernatant was harvested 5 days after transfection.

The HA1 antigen was purified from the supernatant using ANTI-FLAG M2 Affinity Gel (A2220; Sigma-Aldrich/Merck, St. Louis, Missouri, USA) as recommended by the manufacturer. The protein was eluted using 100 µg/mL FLAG peptide (Cat. No. F3290; Sigma-Aldrich/Merck, St. Louis, Missouri, USA). Subsequently, the peptide was removed from the preparation by dialysis against an excess of 1 × PBS, pH 7.4. The same procedure was used for the purification of the full-length H5, which was assessed as an alternative capture antigen in the ELISA.

For H5-Nano, the crude supernatant was harvested, aliquoted, and stored at −80°C until further use.

### Commercial ELISAs

The ID Screen Influenza A Antibody Competition Multi-species ELISA (FLUACA-5P; Innovative Diagnostics, Grabels, France), subsequently termed NP-ELISA, was performed according to the instructions of the manufacturer. Cut-off values were applied as recommended: inhibition ≤45%, positive; 45%–50%, doubtful; ≥50%, negative.

The ID Screen Influenza H5 Antibody Competition 3.0 Multi-species ELISA (FLUACH5V3-5P; Innovative Diagnostics, Grabels, France), subsequently termed ID Screen H5 ELISA, was performed according to the instructions of the manufacturer. Cut-off values were applied as recommended: inhibition ≤50%, positive; 50%–60%, doubtful; ≥60%, negative. Some samples from birds and carnivores were tested in the previous version of this ELISA (ID Screen Influenza A Antibody Competition Multi-species ELISA).

### Samples used for the validation of the ELISA and receiver operating characteristic (ROC) analysis

For ROC analysis, the negative status of samples was defined by a negative result in the ID Screen NP-ELISA. For pigs, a negative status was defined by a negative result in the ID Screen H5 ELISA. Positive samples were tested positive in both the ID Screen H5 ELISA and the NP-ELISA (with the exception of samples from animals vaccinated with DIVA-compatible vaccines, which were tested only for H5-specific antibodies). Samples with discordant results were excluded from statistical analysis but used for comparative evaluations of the ELISAs.

#### Chickens

A total of 296 H5 clade 2.3.4.4b antibody-negative and -positive serum or plasma samples were obtained during immunization and/or infection studies at the Friedrich-Loeffler-Institut (FLI), Germany.

#### Wild birds and zoo birds

Samples were collected in the course of an outbreak of H5N1 in a zoo in Karlsruhe (*n* = 316), Germany, in 2022 (34), or were taken from routine diagnostic submissions to the German National Reference Laboratory (NRL) for avian influenza at the FLI (*n* = 64).

#### Geese

One hundred eighty-five positive and negative serum and plasma samples from geese were obtained during a vaccination study performed at the FLI and collected either before prime immunization, 4 weeks after prime immunization, or 3 weeks after booster vaccination (35).

#### Cattle

Seven positive bovine samples were taken from animal trials performed at the FLI (36). A pool of these samples was used as a positive control for the ELISA. Samples that tested negative for anti-influenza A virus antibodies using an NP-specific ELISA (*n* = 383) were collected during a previous monitoring study and provided by the German NRL for Bovine Viral Diarrhea (37). A pool of several of these samples served as a negative control for the NanoLuc ELISA.

#### Swine

Serum samples from domestic pigs (*n* = 263) and wild boars (*n* = 48) tested negative for H5-specific antibodies using the ID Screen H5 ELISA were collected in Germany during 2009–2010 in the frame of previous studies (38, 39). Samples from wild boars that were previously tested H5 antibody-positive (*n* = 6) were taken from wildlife disease monitoring studies 2024/2025 in Germany (K. Wernike, unpublished data).

#### Ferrets

Anti-H5 antibody-positive samples (*n* = 6) were obtained during infection studies using H5N8 (H5 clade 2.3.4.4b) at the FLI. Antibody-negative samples (*n* = 64) were submitted to the NRL for SARS-CoV-2 infections in animals at the FLI for serological investigations in the frame of routine health monitoring in animal facilities.

### Samples used for the evaluation of the ELISA in comparison with the commercial H5 ELISA

These samples were not included in the ROC analysis but were used to analyze the performance of the ELISA after selection of the cut-off value. An antibody-positive or antibody-negative status was defined as described above. Samples with discordant results in the different ELISAs were re-tested by serum neutralization test (SNT).

#### Wild birds

Fifty-seven samples from wild birds (waterfowl and non-waterfowl) were provided by the Erasmus Medical Centre (EMC), Rotterdam, The Netherlands, from ongoing wild bird surveillance studies.

#### Ferrets

Eight H5 (clade 2.3.4.4b) antibody-positive samples were obtained in the frame of an infection study performed at the FLI.

#### Sheep

Seven hundred fourteen serum samples from routine diagnostic submissions were provided by the State Office for Agriculture, Food Safety, and Fisheries Mecklenburg-Western Pomerania. The samples tested negative for anti-influenza A virus antibodies using the NP-specific ELISA. Samples with discordant results in the H5-specific ELISAs were additionally tested by SNT.

#### Human serum samples

Eight anti-H5 antibody-positive sera, collected from individuals previously vaccinated with an adjuvanted inactivated split virion vaccine based on A/Indonesia/5/2005 (clade 2.1) and some of whom had also been vaccinated with a similar vaccine based on A/Vietnam/1194/2004 (clade 1) vaccine 2 years earlier, were provided by the EMC, Rotterdam, The Netherlands. Sera were collected with informed consent. The Institutional Research Review Board of Erasmus MC Rotterdam confirmed that the rules laid down in the Medical Research Involving Human Subjects Act (also known by its Dutch abbreviation WMO) do not apply to the reuse of these samples from the biobank for development of diagnostic methods. Two pools of these samples were used to investigate the analytical sensitivity of the NanoLuc ELISA. Sera were additionally collected from 11 individuals after a prime-boost vaccination schedule with an inactivated, adjuvanted surface antigen vaccine based on the A/Astrakhan/3212/2020 (H5N8)-like strain (clade 2.3.4.4b). Samples were provided by the Faculty of Medicine and University Hospital Cologne, Germany. The sera were previously characterized using different serological tests (40).

### Samples used to investigate the cross-reactivity of the ELISA

These samples were not included in the ROC analysis. All samples tested positive in the NP-specific ELISA.

#### Chickens

Samples from chickens infected with H5N1 clade 2.2, H5N1 subclade 2.2.2, H6N2, H2N5, H2N9, or H1Nx (*n* = 22) were obtained during infection studies at the FLI, Germany. Samples from chickens vaccinated with a recombinant Newcastle disease virus expressing H5 clade 1 (*n* = 122, before and after homologous challenge infection) and from chickens vaccinated with a recombinant herpesvirus of turkey-vectored vaccine expressing H5 clade 2.2 (*n* = 36) were collected in the frame of vaccination studies performed at the FLI (M. Murr, unpublished data).

#### Ferrets

Samples from animals infected with H2N2 (*n* = 4) were provided by the EMC, Rotterdam, The Netherlands (41). Further samples from ferrets infected with H7N7 (*n* = 3), H7N9 (*n* = 3), H9N2 (*n* = 18), or H5N8 (*n* = 7) were collected during infection studies performed at the FLI.

### Samples from wild terrestrial carnivores

Blood samples from red foxes, raccoons, raccoon dogs, European badgers, and European pine martens (*n* = 628) were collected in 2024 and 2025 in the course of a wildlife disease monitoring project in Mecklenburg-Western Pomerania, Germany (42). All samples were used for the comparative evaluation of the H5-specific ELISAs, and a subset was additionally tested by SNT. Negative samples were determined by a negative result in the NP-ELISA, positive samples by a positive result in both the NP- and H5-specific ID Screen ELISA. Only these defined samples were used for ROC analysis to select the cut-off value for the modified ELISA protocol. Samples with discordant results were excluded from statistical analysis but used for the comparative evaluation of the serological assays.

Further details on all samples used in the study are provided in Table S1 and are available upon request.

### H5-nanoluciferase (H5-NanoLuc) ELISA

The H5-Nano detector antigen was expressed in Expi293 cells as described above. Cell culture supernatant was harvested 5 days after transfection and stored at −80°C without further processing. After first thawing, the aliquots were stored at 4°C for up to 4 weeks. The luciferase activity of the detector antigen per microliter of supernatant was quantified using the Nano-Glo luciferase assay system (#N1110, Promega, Madison, Wisconsin, USA). For this purpose, 10-fold serial dilutions of the supernatant were prepared, mixed with the substrate solution, and the bioluminescence was measured in luciferase light units (LU).

High-binding white flat-bottom ELISA plates (#3922, Corning, New York, USA) were coated with 0.2 µg of the HA1 capture antigen per well (2 mg/mL) in 0.1 M carbonate buffer, pH 9.6, overnight at 4°C. One well on each plate was left uncoated to control the luciferase activity of the applied H5-Nano dilution. Subsequently, the plates were washed three times with washing buffer (1× PBS, 0.1% Tween, pH 7.4) and treated with Liquid Plate Sealer animal-free (#163500, CANDOR Bioscience GmbH, Wangen im Allgäu, Germany) as recommended. After that, the plates were sealed and stored desiccated at 4°C until further use.

For the ELISA, serum samples were diluted 1:10 in dilution buffer (1× PBS, 1% Tween, pH 7.4) in a non-binding microtiter plate (#650101, Greiner Bio-One GmbH, Frickenhausen, Germany). The cell culture supernatant containing the H5-Nano antigen was diluted in dilution buffer to 4 × 10^6^ LU/mL, and 50 µL of this dilution was added to each sample (2 × 10^5^ LU per sample). The mixture of serum and detector antigen was then incubated for 1 h at room temperature (RT). At the same time, the pre-coated ELISA plate was blocked with 5% skim milk in 1× PBS for 1 h at 37°C. Subsequently, the ELISA plate was washed three times with washing buffer, and the pre-incubated sample mixture was added for 1 h at RT. After washing the plate six times with washing buffer, 40 µL of the Nano-Glo substrate solution was added to each well, and the luciferase activity (LU) was measured for 2 s per well using a Tecan Infinite F200 PRO plate reader (Fig. S1A).

### Modifications for samples from wild terrestrial carnivores

For samples collected post-mortem from wild terrestrial carnivores (red foxes, raccoons, raccoon dogs, European badgers, and European pine martens), the original protocol of the NanoLuc ELISA was modified in order to minimize non-specific background signals. The blood samples were mixed 1:10 in dilution buffer and incubated directly on the blocked ELISA plate for 1 h at 37°C, followed by five washes with washing buffer. Subsequently, the H5-NanoLuc detector antigen was diluted in dilution buffer to a concentration of 8–10 × 10^6^ LU/mL, and 50 µL was added to each well for 1 h at RT. The plate was then washed six times with washing buffer, and finally, the luciferase activity was measured as described above (Fig. S1B).

### Serum neutralization test

The serum neutralization test (SNT) was performed according to a published protocol (43). Briefly, the test samples were heat-inactivated at 56°C for 1 h. Twofold dilution series of the samples were prepared in cell growth medium supplemented with 2 µg/mL trypsin on 96-well microplates. The test virus was diluted in the same medium to a concentration of 10^3.3^ TCID_50_/mL, and 50 µL was added to each serum dilution. After incubation for 1 h at 37°C, the serum/virus mixture was transferred to 96-well plates with confluent MDCK-II cells, which had first been washed twice with cell growth medium. The plates were then incubated at 37°C, and after 72 h, the readout was performed by assessing the cytopathic effect. Titers with the reciprocal of neutralization dose 50 (ND50) ≥20 were defined as positive.

The recombinant test virus carrying the HA gene from A/Caspian gull/Netherlands/1/2022 (H5N1, clade 2.3.4.4b) (EPI1967017) (44) and the remaining seven gene segments from the A/Puerto Rico/8/1934 (H1N1; PR8) backbone was rescued by reverse genetics according to reference 45 with slight modifications. Briefly, co-cultures of HEK293T and MDCK-II cells were co-transfected with the eight plasmids encoding the desired gene segments and incubated for 72 h. Supernatants were harvested and inoculated into the allantoic cavity of 10-day-old specific-pathogen-free embryonated chicken eggs (Valo BioMedia GmbH, Osterholz-Scharmbeck, Germany). After 72 h, allantoic fluids were harvested and tested for hemagglutination activity and the absence of bacterial contamination. The virus stock was stored at –80°C until further use.

### Validation methods and statistical analyses

ROC analysis was used for determination of cut-off values and assessment of test sensitivity and specificity. The commercial ID Screen H5 ELISA and NP-ELISAs were used as surrogate reference tests for the analysis since no real gold standard test was available. Samples with discordant results in the two ELISAs were excluded from statistical analyses. Agreement between the ID Screen H5 ELISA and the H5 NanoLuc assay was calculated using the Cohen’s kappa coefficient (“κ”). Samples that were classified as “doubtful” by the commercial ELISA were excluded from calculation of κ. All statistical analyses were performed using GraphPad Prism 10.0.

## RESULTS

### Protein expression and purification

The H5-HA1 subdomain (aa 17–345) used as the capture antigen for the ELISA was expressed in Expi293 cells and FLAG-purified from the supernatant. An optimal coating concentration of 0.2 µg/well (2 mg/mL) was determined by checkerboard titration using defined antibody-positive and -negative cattle and chicken serum samples.

The H5-Nano detector antigen was expressed at high levels, yielding approximately 1 × 10^12^ LU/mL luciferase activity in a single six-well transfection of Expi293 cells, which is sufficient for testing more than 1 × 10^6^ samples. Stored at −80°C, the luciferase activity was completely preserved over at least 2 years. After first thawing, the antigen could be stored at 4°C, and for up to 2 weeks, its luciferase activity remained stable. However, it declined by 20 to 30% after 1 to 2 months and by 60 to 70% after 3 to 4 months of storage at 4°C. Lyophilization of the crude supernatant resulted in a slightly reduced luciferase activity (approximately 30%) but showed no negative impact on the functionality of the antigen. However, no long-term stability studies were performed for this approach within the frame of this study. The limited stability of the detector antigen at 4°C represents a relevant weakness of the NanoLuc ELISA and will need to be optimized to allow a robust application in the field. In its current format, it is crucial to check the luciferase activity of the applied H5-Nano dilution in a separate well of each test in order to maintain reproducibility of the assay.

**Fig 1:**
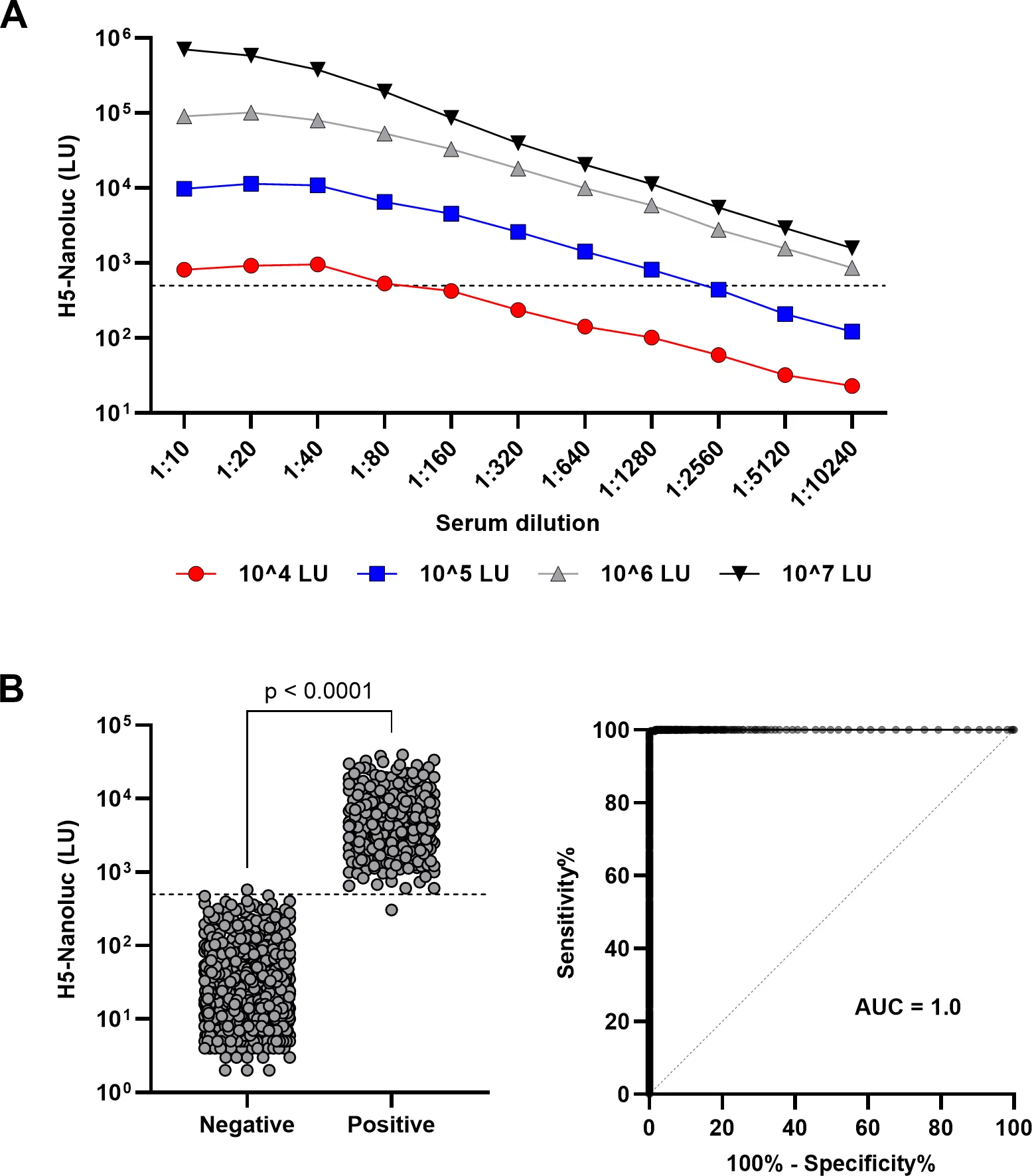
Assay parameters and performance of the H5-NanoLuc assay. (**A**) Dynamic detection range of the H5-NanoLuc assay dependent on the concentration of the detector antigen. Different dilutions of the H5-Nano antigen (10^4^, 10^5^, 10^6^, or 10^7^ LU per sample) were comparatively tested with a serially diluted antibody-positive cattle serum sample. (**B**) Positive (*n* = 342) and negative samples (*n* = 1,096) from different mammalian and avian species showed significantly different ELISA reactivity (unpaired *t*-test, *P* < 0.0001). The cut-off value of ≥500 LU was determined by ROC analysis and is indicated with a dashed line. ROC analysis yielded an area under the curve (AUC) of 1.0 (95% confidence interval [CI] 0.99–1.00; *P* < 0.0001).

To determine the optimal input concentration of the detector antigen in the ELISA, checkerboard titrations were performed using serially diluted antibody-positive cattle serum samples. It became evident that the highest sensitivity with a limit of detection (LOD) of 1:10,240 could be achieved by using 10^6^–10^7^ LU of the detector antigen per sample. Using only 10^5^ or 10^4^ LU of the detector antigen per sample showed a comparatively lower sensitivity with LODs of 1:1,280 and 1:80, respectively (Fig. 1A). In order to achieve an optimal signal-to-noise ratio, the concentration of the H5-Nano detector to be used in the ELISA was adjusted to 2 × 10^5^ LU per sample (4 × 10^6^ LU/mL). Due to the modified incubation strategy, the amount of the detector antigen had to be determined separately for the wild carnivore protocol, and an optimal H5-Nano concentration of 4–5 × 10^5^ LU (8–10 × 10^6^ LU/mL) was selected for this approach.

### Test performance

In order to allow a species-independent application of the ELISA, a pooled panel of positive (*n* = 342) and negative (*n* = 1,096) samples from cattle, pigs, wild boars, ferrets, chickens, and different bird species (waterfowl and non-waterfowl) was used to choose an optimal cut-off value by ROC analysis. The area under the curve (AUC) was determined to be 1.0 (95% CI: 0.99–1.00; *P* <0.0001). For the selected cut-off value of ≥500 LU, the sensitivity was estimated to be 99.71% (CI: 98.36%–99.99%), and the specificity to be 99.82% (CI: 99.34%–99.97%) (Fig. 1B). Since no gold standard test was available for a multispecies application, the positive status of sera was defined by a positive result in both the commercial ID Screen H5 ELISA and the NP-specific ELISA. Samples from individuals vaccinated with DIVA-compatible vaccines were only tested in the H5 ELISA. A negative status was defined by a negative test result in the NP-specific ELISA. For pigs, negative samples were tested negative in the H5 ELISA. Samples with discordant results in the H5- and NP-specific ELISAs were excluded from ROC analysis. However, not all samples included in the analysis were tested in both assays or re-tested by SNT. Thus, bias due to misclassification in the commercial ELISAs cannot be excluded and should be considered for the assessment of diagnostic sensitivity and specificity.

To investigate the within-run variation of the ELISA, one sample was tested 40–60 times on one plate. In three independent runs, the coefficient of variation (CV%) of the sample was between 6 and 18% (corresponding to signal differences between 350 and 660 LUs). In order to determine the between-run variation, 15 samples were tested on 10 different plates on 10 different days. The CV% ranged from 9 to 29% for the individual samples. Higher variations were observed for strongly and weakly positive samples (signal differences between 5,000 and 7,000 LUs and 200 and 1,200 LUs, respectively). For negative samples, variations were between 20 and 130 LUs. These rather high CVs are probably associated with the liquid-phase pre-incubation and the subsequent transfer from one plate to another. Importantly, these variations can lead to a misclassification of weakly positive samples between different runs. For a future application in the field, results with values between 400 and 500 LU should be classified as “doubtful,” requiring confirmation in a second test. However, this was not considered during the present study. As the sample quality had a significant impact on its performance, it was not possible to evaluate the repeatability of the modified ELISA protocol used for terrestrial carnivore samples.

We also examined the cost-effectiveness of the assay with regard to its potential for large-scale testing. Based on the required materials and the necessary infrastructure, including protein expression and purification, the costs were estimated to be approximately 60 € for one plate of 88 samples; that is, about 70 cents per sample, which is consistent with commercially available ELISA kits. For the ID Screen NP-ELISA or H5 ELISA used in this study, one sample costs between 1 and 2 €.

### Detection of H5-specific antibodies in different bird species

**Fig 2:**
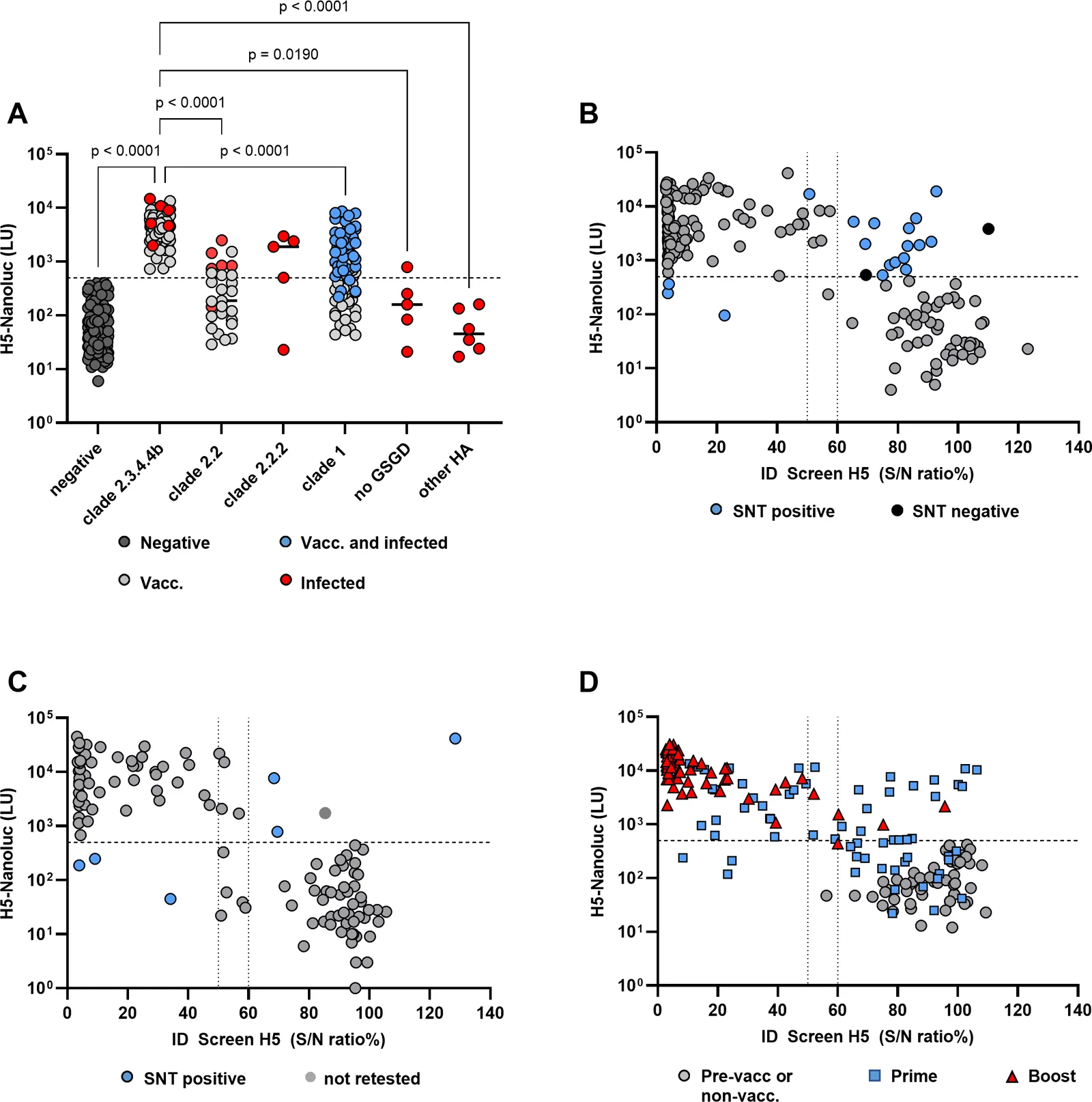
Performance of the NanoLuc ELISA for samples from avian species. (**A**) Samples from chickens vaccinated and/or infected with the indicated H5 clades/subclades. Vaccinated animals are shown in gray, infected animals in red. Animals that were vaccinated and subsequently infected are highlighted in blue. “No GSGD” indicates infection with an H5 strain not belonging to the A/goose/Guangdong/1/96 lineage. “Other HA” included antibodies against subtypes H1, H2, and H6. All negative samples were tested negative for anti-influenza A virus antibodies using the NP-specific ELISA. Differences between groups were analyzed by Kruskal-Wallis test followed by Dunn’s multiple comparisons test. Only significant differences (*P* ≤ 0.05) between individual groups vs the clade 2.3.4.4b group are shown on the graph. Samples from waterfowl (**B**) and from non-waterfowl (**C**) bird species were tested comparatively with the two H5-specific ELISA formats. Results of the ID Screen H5 ELISA (in S/N% ratio) are plotted on the *x*-axis, and the cut-off values are indicated with dotted lines. Results of the NanoLuc assay (in LU) are plotted on the *y*-axis; the cut-off value is indicated with a dashed line. Samples colored in blue were additionally tested positive by SNT, samples in black were negative. (**D**) Serum samples from gees collected in the context of vaccination study. The samples were obtained from non-vaccinated animals and from animals before and after prime and booster immunization with an H5 clade 2.3.4.4b-specific vaccine. The graph is structured as described for panels **B** and **C**.

H5 clade 2.3.4.4b antibody-positive samples obtained from vaccinated (*n* = 102) or infected (*n* = 6) chickens were used for the initial evaluation of the H5-Nano ELISA, and the test allowed a clear and significant (*P* < 0.0001) distinction between positive and negative samples with a broad dynamic detection range (Fig. 2A). Additionally, antibodies were detected in chickens after infection with H5N1 of clade 2.2 or subclade 2.2.2. However, in chickens that were vaccinated with a recombinant herpesvirus of turkey-vectored vaccine expressing an H5 subtype of clade 2.2, the H5-Nano assay reacted only with 4 out of 21 samples, which were determined to be H5 antibody-positive by the ID Screen ELISA. A clearly inferior performance was also observed for the detection of antibodies against H5 of clade 1. In chickens vaccinated with a live Newcastle disease virus-vectored vaccine expressing an H5 of clade 1, the ELISA detected 18 out of 41 positive animals, and only after homologous challenge infection with H5N1 (A/ck/Viet/P41/05) >90% of the samples were correctly recognized. Not surprisingly, antibodies of animals infected with a low pathogenicity H5N2 strain (A/duck/Potsdam/1402-6/1986 [H5N2]), not belonging to the A/goose/Guangdong/1/96 lineage, were detected only in one out of five animals. Furthermore, no cross-reactivity was observed with antibodies against the HA of subtypes H1, H2, and H6 in samples from experimentally infected chickens. Even though subtypes H1, H2, and H6 are phylogenetically more closely related to H5 than other subtypes, the measured LU values did not significantly differ from the ones obtained for H5-negative samples (Fig. 2A).

In addition to galliform poultry, the performance of the NanoLuc ELISA was investigated for several other wild and zoo bird species. One hundred ninety-three samples were obtained from wild or captive waterfowl, including different duck, goose, and swan species (Fig. 2B). H5-specific antibodies were detected in 123 and in 120 samples by the ID Screen and the NanoLuc H5 ELISA, respectively. Interestingly, among the 65 samples showing reproducibly negative results in the commercial ELISA, several samples reacted positive in the NanoLuc assay (*n* = 17; 11 swans, 2 ducks, 4 geese). Based on this outcome, a substantial agreement of the two ELISA formats (Cohen’s kappa coefficient κ = 0.752, 95% CI: 0.651–0.853) was determined. However, when samples with inconsistent results were additionally tested in an SNT, neutralizing antibodies were detected in 15 out of the 17 samples yielding positive results in the NanoLuc ELISA, which suggests a superior sensitivity compared to the commercial test.

In addition to waterfowl, 122 samples from other wild bird species (gulls, terns, waders, raptors) and captive zoo birds (e.g., peafowl, flamingo, pelicans, cranes, marabou, toucan, ostriches, penguins, owls, macaws, cockatoos, and pigeons) were investigated (Fig. 2C). The ID Screen H5 and the NanoLuc ELISA detected anti-H5 antibodies in 59 and 56 samples, respectively. Overall, discordant results were observed only for seven samples (pelicans or gulls), which indicated an almost perfect agreement (κ = 0.976, 95% CI: 0.787–0.965) between the two assays for samples from non-waterfowl bird species.

The suitability of the ELISA to detect antibodies induced upon vaccination was investigated using samples obtained in the frame of two immunization studies in geese (35) (Fig. 2D). Within this panel, the agreement of the NanoLuc assay with the commercial ID Screen H5 ELISA was found to be substantial (κ = 0.650, 95% CI: 0.522–0.777). Notably, discordant results were mainly observed for sera collected after prime but before booster immunization, showing positive results in the NanoLuc ELISA, whereas the ID Screen ELISA still scored negative (*n* = 17).

### Detection of H5-specific antibodies in mammalian species and humans

**Fig 3:**
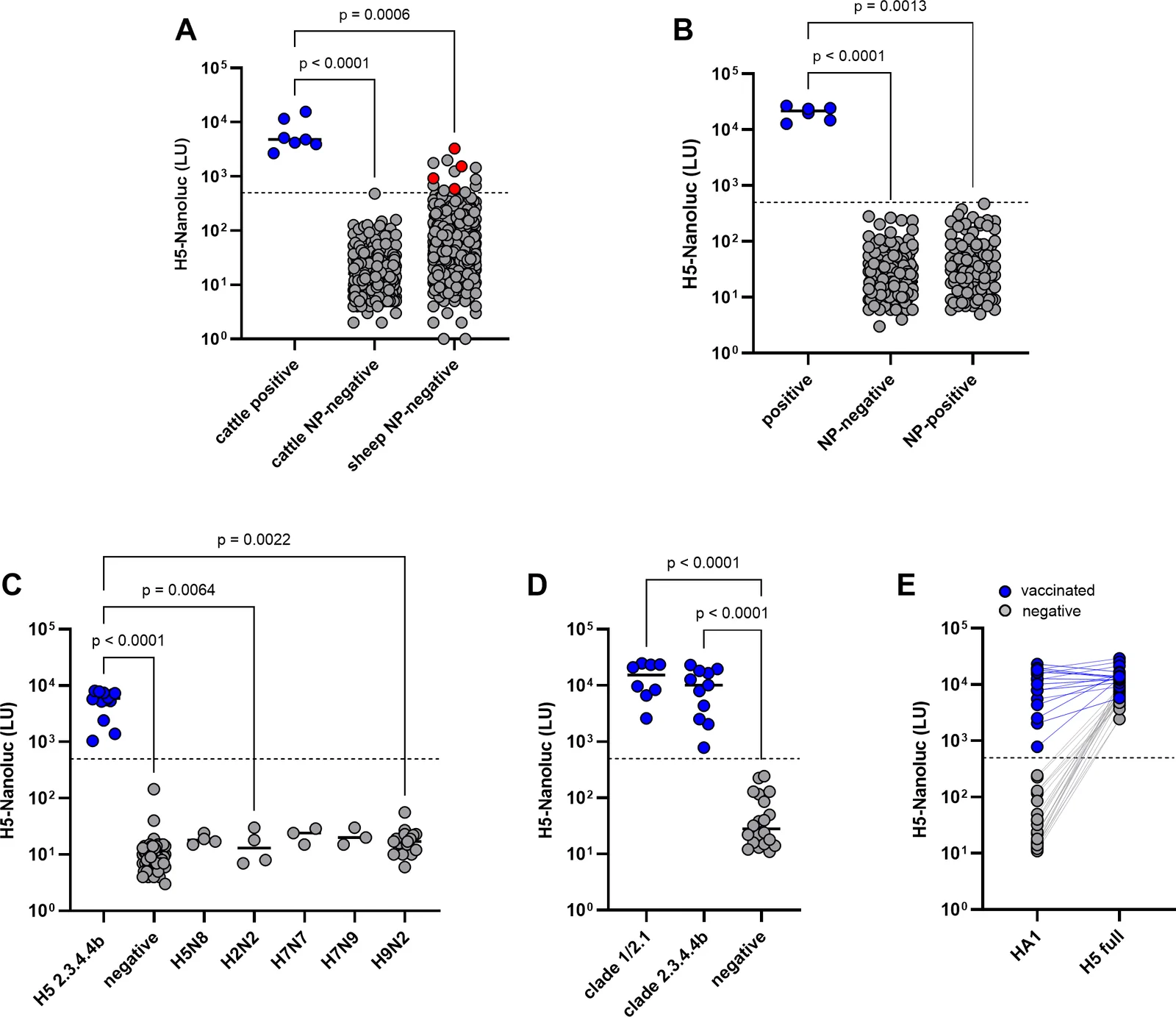
Performance of the NanoLuc ELISA for samples from mammalian species and humans. (**A**) Samples from H5N1-infected cattle and samples from cattle and sheep that were tested negative for anti-influenza A NP-specific antibodies. Samples from sheep marked in red were tested positive in the commercial ID Screen H5 ELISA and/or by SNT. Statistical differences were calculated using the Kruskal-Wallis test followed by Dunn’s multiple comparisons test. (**B**) Samples from domestic pigs and wild boars. Positive samples tested positive with the ID Screen H5 ELISA. NP-negative samples were tested negative in the ID Screen NP-ELISA, NP-positive samples tested positive. Statistical differences were calculated using the Kruskal-Wallis test followed by Dunn’s multiple comparisons test. (**C**) Samples from ferrets infected with influenza A viruses of the indicated HA subtypes. Negative samples were tested negative for NP-specific antibodies. Statistical differences were calculated using the Kruskal-Wallis test followed by Dunn’s multiple comparisons test. Only significant differences (*P* ≤ 0.05) vs the H5 clade 2.3.4.4b group are shown on the graph. (**D**) Human serum samples from H5N1-naïve individuals and individuals vaccinated with H5-specific vaccines. Vaccine antigens were based on H5 clade 1/clade 2.1 (*n* = 8) or H5 clade 2.3.4.4b (*n* = 11). All antibody-positive samples were also tested positive for neutralizing antibodies by SNT. Statistical differences were calculated using the Kruskal-Wallis test followed by Dunn’s multiple comparison tests. (**E**) Differentiation of H5 head-specific from cross-reacting anti-stalk antibodies in human serum samples using either the HA1 subdomain or the full-length H5 as a capture antigen in the ELISA. H5-naïve individuals are shown in gray, individuals vaccinated with H5-specific vaccines in blue. In all graphs, the cut-off value of the NanoLuc ELISA (≥500 LU) is indicated with a dashed line.

In sera of cattle infected with H5N1 clade 2.3.4.4b (36), the NanoLuc ELISA detected H5-specific antibodies starting from 9 days after infection, with a significant distinction (*P* < 0.0001) from negative samples (Fig. 3A). Furthermore, the assay demonstrated superior analytical sensitivity since the LOD for three different sera was markedly below that of the ID Screen H5 ELISA. Specifically, the LOD of the H5-NanoLuc assay vs the commercial test was found to be 1:80 vs 1:40 for cattle #1, 1:160 vs 1:10 for cattle #2, and 1:640 vs 1:80 for cattle #3 (Fig. S2A). Since no background or non-specific signals were observed during testing of influenza A virus antibody-negative cattle sera, an additional 714 NP-ELISA-negative sera from sheep were tested to probe the test specificity. Among these, 14 samples yielded positive results in the H5 NanoLuc ELISA, resulting in a relative test specificity of 98% (compared to the NP-ELISA), which is close to the 99.82% predicted by ROC analysis. However, bias due to misclassifications by the NP-ELISA has to be considered since two of the supposedly false-positive samples also tested positive using the ID Screen H5 ELISA, and two others showed neutralizing activity in an SNT (Fig. 3A).

Among samples from domestic pigs and wild boars, high levels of H5-specific antibodies were detected in six wild boar samples collected in 2024 in Germany, whereas no antibody-positive animals were found in the panels obtained between 2009 and 2010. Interestingly, in about 40% (104/263) of the sera originating from domestic pigs, anti-influenza A virus antibodies were found by the NP-ELISA. However, none of these sera reacted positive in the NanoLuc assay, which underscores its high specificity for H5-specific antibodies (Fig. 3B). This finding was confirmed by testing samples from ferrets experimentally infected with different influenza A virus strains. Antibodies raised upon infection with H5 clade 2.3.4.4b strains reacted highly positive, whereas no signals were observed for naïve ferrets and animals that were infected with H5N8, H2N2, H7N9, H7N7, or H9N2 (Fig. 3C).

Human samples collected from individuals immunized with H5-specific vaccines (antigens based on H5 clade 1/clade 2.1 or on H5 clade 2.3.4.4b) reacted highly positive in the NanoLuc assay (Fig. 3D). Testing dilution series of two antibody-positive serum pools determined a LOD of 1:160 for the NanoLuc ELISA compared to an LOD of 1:10 in the commercial ELISA, which demonstrates a superior analytical sensitivity of the novel assay (Fig. S2B). All serum samples from H5N1-naïve individuals were tested negative, despite containing H1- and H3-specific antibodies (40). In addition, the previously identified cross-reactive antibodies that target the HA stalk domain (40) were not detected using the HA1 capture antigen. However, their presence was confirmed when the full-length H5 was used as the capture antigen (Fig. 3E). Thus, testing samples with both antigens in parallel offers the possibility to differentiate cross-reacting anti-stalk antibodies from H5 head-specific antibodies.

**Fig 4:**
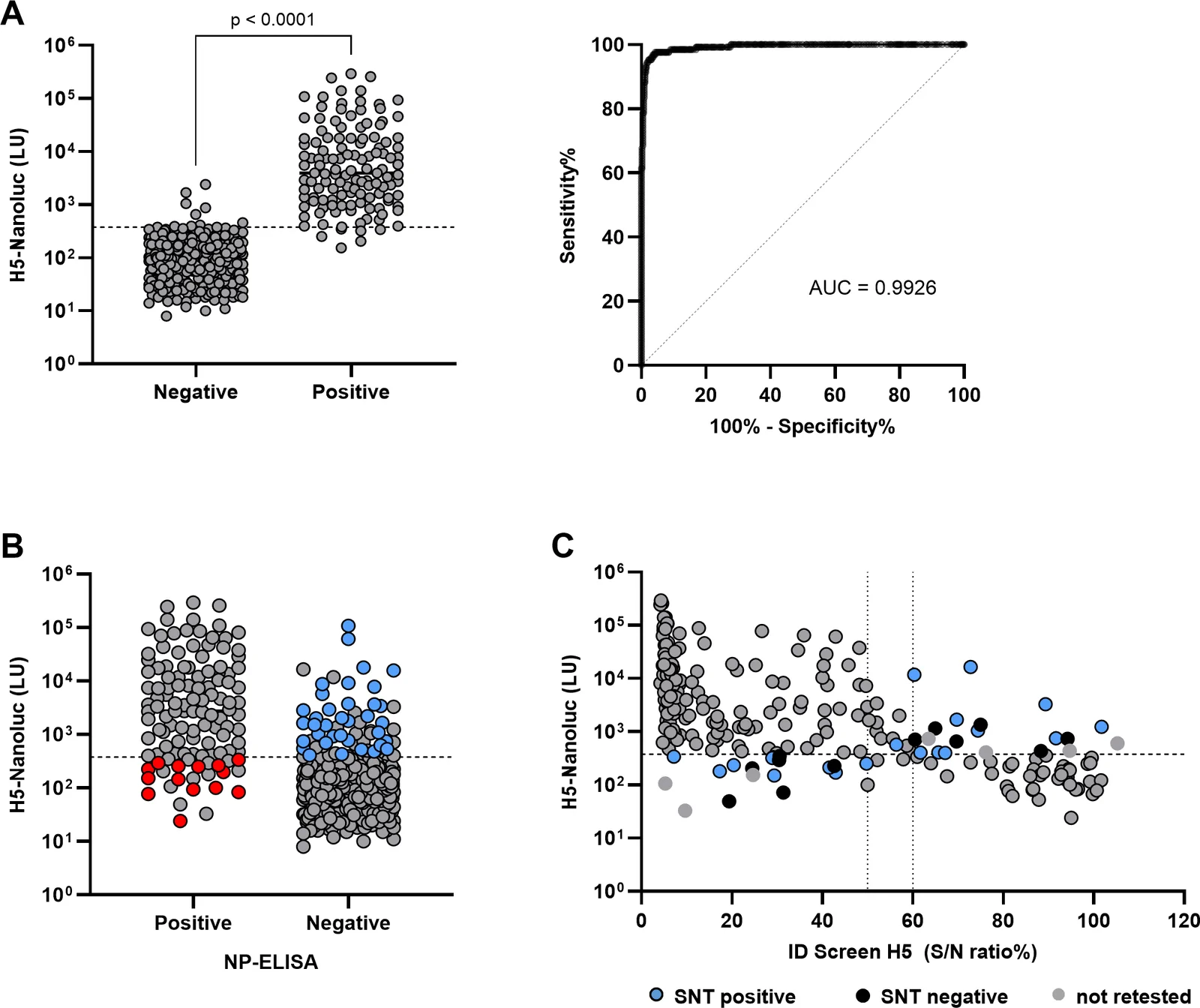
Performance of the NanoLuc ELISA for samples from wild terrestrial carnivores. (**A**) Positive (*n* = 127) and negative (*n* = 443) samples from different wild terrestrial carnivores showed significantly different ELISA reactivity (*P* < 0.0001, unpaired *t*-test). The cut-off value of ≥380 LU was determined by ROC analysis and is indicated with a dashed line. ROC analysis yielded an AUC of 0.9926 (95% CI: 0.9867–0.9984, *P* < 0.0001). (**B**) Detection of H5-specific antibodies by the NanoLuc ELISA in samples pre-tested for anti-influenza A virus antibodies using the commercial NP-ELISA. The cut-off value is indicated with a dashed line. Samples highlighted in red were additionally tested positive in the ID Screen H5 ELISA; samples in blue were tested negative. (**C**) Comparison of the H5-NanoLuc and the ID Screen H5 ELISA for samples tested in both assays in parallel. Results of the ID Screen H5 ELISA (in S/N ratio %) are plotted on the *x*-axis, and the cut-off values are indicated with dotted lines. Results of the NanoLuc assay (in LU) are plotted on the *y*-axis; the cut-off value is indicated with a dashed line. Samples colored in blue were tested positive by SNT; samples in black were negative.

For samples obtained post-mortem from wild terrestrial carnivores, it was necessary to modify the original ELISA protocol due to high non-specific background signals. This problem was overcome by incubating the diluted samples on the coated plate first, followed by a washing step and subsequently adding the H5-Nano detector antigen instead of performing the original liquid-phase co-incubation. Positive (*n* = 127; positive in both, H5- and NP-specific ID Screen ELISA) and negative (*n* = 443; negative in the NP-specific ELISA) samples from red foxes, raccoons, raccoon dogs, European badgers, and European pine martens (42) were used to evaluate the diagnostic performance of this adapted protocol in comparison to the commercially available ELISA. ROC analysis was performed using the commercial ID Screen ELISAs as reference tests and yielded an AUC of 0.9926 (95% CI: 0.9867%–0.9984; *P* <0.0001). Applying a cut-off value of ≥380 LU, the test sensitivity was estimated at 95% (CI: 90.08%–97.82%) and the specificity at 98% (CI: 96.18%–98.93%) (Fig. 4A). Additional assay parameters, such as repeatability and reproducibility, were not investigated for this modified protocol due to the limited volume and the poor quality of the available samples.

Upon screening with the NP-specific ELISA, 121 out of 619 carnivore samples tested positive for influenza A virus NP-specific antibodies (Fig. 4B). The NanoLuc assay subsequently detected H5-specific antibodies in 79% of these samples (96/121). Possibly, antibodies in the remaining samples were directed against other HA subtypes since they were mostly also classified as negative or doubtful by the ID Screen H5 ELISA. Interestingly, several anti-NP-negative samples reacted positive in both the NanoLuc and the ID Screen H5 ELISA, which indicates a superior sensitivity of these assays for the detection of subtype-specific antibodies and a potential misclassification by the NP-ELISA (Fig. 4B). However, when the test results of 215 samples examined in both H5 ELISAs were directly compared, only a moderate agreement was determined (κ = 0.532, 95% CI: 0.403–0.661) and for several samples, clear discrepancies became obvious (Fig. 4C). In order to investigate this in more detail, we additionally analyzed these samples by SNT. Comparing the results of the three different ELISAs and the SNT revealed inconsistencies across all assays, implying that the reduced quality of the test material obtained from wild carnivores had a considerable impact on the test performance. These findings make it difficult to assess the true diagnostic sensitivity and specificity of the individual assays (Table S2).

## DISCUSSION

The ongoing panzootic demonstrates an unprecedented spread and rapid evolution of the H5N1 HPAI virus, with numerous outbreaks and massive die-offs in wild and domestic animals never reported before. Rising numbers of spillover infections in mammals indicate an elevated risk of zoonotic transmission, emphasizing the need for continued monitoring and targeted preparedness measures. Thus, increased serological surveillance is critical to identify new host species and to uncover potential paths of silent spread. For this purpose, robust assays with no restriction to selected species are urgently needed.

In the present study, a novel H5-specific ELISA was developed based on the previously described LIPS technology, which has proven to be a highly sensitive antibody detection method for diagnosis of a broad variety of autoimmune and infectious diseases (46, 47). A LIPS assay for detection of H5-specific antibodies using HA1 and HA2 detector-antigens has previously been described (48). However, the original LIPS platform is based on the binding of antibody-antigen complexes to protein G beads, which is not applicable for all species, most notably from avian origin. In contrast, the double-antigen ELISA format allows a species-independent detection of antibodies against influenza A virus, as it has been shown before (49). However, the validation of such a broadly applicable test is challenging due to the lack of reference samples and gold standard tests for many species, especially wildlife (50, 51). Although the commercial H5- and NP-specific ELISA have not been validated for multispecies applications, we decided to use them as reference tests to evaluate the H5-NanoLuc assay. Thus, we cannot exclude bias in the assessment of diagnostic sensitivity and specificity, which represents a critical limitation of our validation data.

Among several previously developed ELISAs, variations of sensitivity, specificity, and accuracy have been described for different bird species, in particular among waterfowl (18, 23, 52, 53). The H5-NanoLuc ELISA allowed a sensitive, specific, and homogeneous detection of H5-specific antibodies in a broad variety of bird species in good agreement with the ID Screen H5 ELISA. However, for samples from ducks, geese, and swans, the NanoLuc assay demonstrated a superior sensitivity compared to the commercial ELISA. One possible explanation for this finding could be the distinct antibody repertoire in non-galliform birds. In addition to the full-length IgY antibody, ducks additionally produce a smaller version of IgY lacking the Fc region (IgYΔFc), which becomes predominant in the serum at later stages of the immune response (54, 55). Using the double-antigen ELISA format, antibodies are detected by simultaneous binding to their specific antigen, which means that all immunoglobulin isotypes, including IgYΔFc fragments, can be efficiently captured. In the competitive ID Screen ELISA, the smaller IgY versions might not be able to compete sufficiently with the labeled detector antibody, which can lead to false-negative test results. The superior performance of the NanoLuc ELISA for samples from waterfowl was further underscored by testing samples from vaccinated geese. In several birds, the assay detected antibodies already after prime immunization, whereas the ID Screen ELISA mostly yielded negative results before the booster vaccination. Thus, the NanoLuc format is a promising tool in the context of future H5-specific vaccination programs in poultry since highly sensitive and high-throughput serological assays will be required to assess vaccine coverage in herds immunized with DIVA-compatible vaccines. However, for surveillance studies, it has to be considered that the high sensitivity of the test might cause a certain amount of false-positive test results. Thus, positive reactions of samples tested negative for anti-influenza A virus antibodies in an NP-specific ELISA should be assessed carefully.

With the emergence of H5N1 2.3.4.4b viruses, serological screening and monitoring of mammalian species have become increasingly important due to the frequent spillover events and adaptation to new hosts (1, 2). The NanoLuc ELISA showed a good diagnostic performance for samples from cattle, domestic pigs, wild boars, ferrets, and humans. Particularly for samples from cattle and vaccinated humans, the newly developed assay demonstrated a superior analytical sensitivity and lower limits of detection compared to the commercial H5 ELISA. This could be explained by the fact that different species use varying strategies to create their individual antibody repertoires (56). Consequently, some species might develop antibodies preferentially targeting epitopes on H5 that are not covered by the detector antibody in the competition ELISA. In contrast, the liquid-phase NanoLuc ELISA allows binding of antibodies to all accessible epitopes on the antigen, independent of species-specific variations. Accordingly, the test was suitable for the detection of H5-specific antibodies in several wild carnivore species but only in moderate agreement with the ID Screen ELISA. However, the respective samples were collected postmortem from carcasses on which autolytic processes had already started, and the discordant test results are likely caused by assay-specific susceptibilities to certain inhibitors. This also explains the necessity to modify the protocol of the H5-NanoLuc ELISA. The lack of suitable reference samples and a true gold standard test makes the interpretation of these data and the observed disagreements challenging. Additional analysis of the samples by SNT revealed further inconsistencies between all serological assays. This confirms that the quality of the sample matrix has indeed an impact on the test performance, independent of the antigen or test format. These results suggest that using the two H5-specific ELISAs in combination can compensate for weaknesses of each assay, making it the most promising strategy to determine the prevalence of H5-specific antibodies also among wild carnivore populations.

The NanoLuc ELISA demonstrated high specificity for antibodies against H5 of clade 2.3.4.4b and, although we did not perform a systematic investigation, some of our data indicate an insufficient sensitivity to detect antibodies against certain other H5 clades. It has previously been observed that H5 clade 2.3.4.4b-specific antibodies from vaccinated or infected mice, as well as from vaccinated humans, bind strongly to the homotypic antigen but only weakly to H5 proteins of other clades (57). However, this clade-optimized antibody detection represents a relevant limitation of the assay in the context of a broader H5 surveillance and its application in areas where viruses of different clades may be co-circulating. In such cases, a combination with the more broadly reactive ID Screen ELISA should be considered. Furthermore, it will be crucial to continuously adapt and update the ELISA antigens to match virus strains that are appearing in the region of interest.

It also became obvious that the selection and design of the capture and detector antigens play a critical role in the specificity of the NanoLuc assay. Using the full-length H5 as a detector antigen allows binding and detecting of antibodies elicited against both the H5 head and stalk domains. However, in combination with HA1 serving as the capture antigen, only antibodies specifically targeting the H5 head domain can be detected, which requires exposure to this particular antigen. As a result, the NanoLuc ELISA did not react with antibodies against HA subtypes H2 and H6, whereas this could repeatedly be observed in previously developed assays (19, 29). This specificity was further underscored by the lack of cross-reactivity with antibodies against H7 or H9 in experimentally infected ferrets or against H1 and H3 in samples from humans or pigs. Based on that finding, the NanoLuc ELISA is an excellent tool for the detection of past H5 infections in pigs. Even though previous studies described a low susceptibility of pigs to infections with H5N1, including the bovine-derived H5N1 B3.13 (58, 59), they represent a potential host for the generation of novel virus reassortants with a possible pre-pandemic risk. Thus, increased surveillance using a highly sensitive and specific serological test is crucial for early awareness (16). The H5-NanoLuc ELISA could further be helpful for monitoring future vaccination strategies with an H5-specific vaccine in humans (60). In previous studies, it was shown that broadly cross-reacting antibodies directed against the conserved HA stalk domain are produced following vaccination with seasonal influenza vaccines. Consequently, detectable levels of cross-neutralizing antibodies can also be found in individuals who have not been exposed to H5N1 (40, 61, 62). Using the HA1 subdomain as a capture antigen, these pre-existing anti-stalk antibodies are not detected by the NanoLuc ELISA. Therefore, the assay can be used to verify the generation of anti-H5 antibodies after H5 vaccination as well as to identify H5-naïve individuals.

### Conclusions

We could show that the newly developed, luciferase-based double-antigen ELISA allows sensitive and specific detection of H5-specific antibodies in a broad range of avian and mammalian species, including humans. Compared to the commercially available ID Screen H5 ELISA, it showed superior performance with regard to multispecies application. As the assay detects only antibodies targeting the H5 head domain, it is suitable for H5-specific surveillance even in species with previous exposures to other HA subtypes. Furthermore, the possibility to differentiate H5 head- from stalk-specific antibodies allows the identification of H5-naïve individuals, which is a highly valuable feature in the context of future monitoring and vaccination programs with H5-specific vaccines. Therefore, in combination with the commercially available ELISA formats, the NanoLuc assay would markedly improve serological diagnostic tools for sensitive detection of H5-specific antibodies in a broad range of species.

## SUPPLEMENTAL MATERIAL

Figure S1 jcm.01907-25-s0001.tif Assay concept.

Figure S2 jcm.01907-25-s0002.tif Analytical sensitivity.

Tables S1 and S2 jcm.01907-25-s0003.docx Description and characterization of samples.

ASM does not own the copyrights to Supplemental Material that may be linked to, or accessed through, an article. The authors have granted ASM a non-exclusive, world-wide license to publish the Supplemental Material files. Please contact the corresponding author directly for reuse.
