# Supplementary material for: Development of a multi-species luciferase-based double-antigen ELISA for the detection of antibodies against influenza A virus H5 clade 2.3.4.4b
Source: J Clin Microbiol. 2026 Aug 13;64(9):e01907-25. doi: 10.1128/jcm.01907-25 (PMC13556203; doi:10.1128/jcm.01907-25)
Supplement: Supplementary file 1 [file jcm.01907-25-s0003.docx]

| **Species** | **Number** | **H5 antibody status** | **H5 infection / vaccination status** | **Source of samples** |
| --- | --- | --- | --- | --- |
| Chickens | 6 | positive | infected with H5N1 (A/chi/Ger-SH/AI08298/2021), H5 clade 2.3.4.4b | Infection studies performed at the FLI |
|  | 6 | positive | infected with H5N1 (A/chi/Viet/P41/05), clade 2.2 |  |
|  | 5 | positive | infected with H5N1 (A/Cygnus cygnus/Germany/R65/2006), subclade 2.2.2 |  |
|  | 5 | positive | infected with A/duck/Potsdam/1402-6/1986 (H5N2), no GSGD |  |
|  | 102 | positive | vaccinated with a recombinant Newcastle Disease Virus (NDV) expressing H5 clade 2.3.4.4b and H5 clade 1 | Vaccination study performed at the FLI |
|  | 122 | positive / negative | vaccinated with a recombinant NDV expressing an H5 clade 1 and infected with H5N1 (A/chi/Viet/P41/05) | Vaccination and challenge infection study performed at the FLI |
|  | 36 | positive / negative | vaccinated with a recombinant herpesvirus of turkey (HVT)-vectored vaccine expressing an H5 of clade 2.2 | Vaccination study performed at the FLI |
|  | 188 | negative | naïve, non-infected or non-vaccinated | Collected from trials mentioned above |
|  | 6 | negative | infected with H6N2, H2N5, H2N9 or H1Nx | Infections studies performed at the FLI |
| Zoo birds | 316 | positive / negative | infected with H5N1 / naïve | Zoo Karlsruhe, Germany (34) |
| Gulls, swans | 20, 44 | positive / negative | infected with H5N1 / naïve | Routine diagnostic submission to the NRL for AI at the FLI |
| Wild birds | 57 | positive / negative | infected with H5N1 / naïve | Provided by the Erasmus Medical Center (EMC), Rotterdam, The Netherlands from ongoing wild bird surveillance studies. |
| Geese | 185 | positive / negative | vaccinated with an H5-specific vaccine, H5 clade 2.3.4.4b | Vaccination study performed at the FLI (35) |
| Ferrets | 6 | positive | infected with H5N8 clade 2.3.4.4b | Infection study performed at the FLI (unpublished data) |
|  | 8 | positive | infected with H5N1 clade 2.3.4.4b | Infection study performed at the FLI (unpublished data) |
|  | 4 | negative | infected with H2N2 | Provided by the EMC, Rotterdam, The Netherlands (41) |
|  | 64 | negative | naïve | Routine diagnostic submissions to the NRL for SARS-CoV-2 infections in animals at the FLI |
|  | 28 | positive / negative | infected with H7N7, H7N9, H9N2, H5N8 | Infection studies performed at the FLI. |
| Sheep | 714 | negative | naïve | Routine diagnostic submissions to the State Office for Agriculture, Food Safety and Fisheries Mecklenburg-Western Pomerania 2024 and 2025 |
| Cattle | 7 | positive | infected with H5N1 | Infection study performed at the FLI (36) |
|  | 383 | negative | naïve | Surveillance study (37) |
| Domestic pigs | 159 | negative | naïve | Surveillance study (38) |
|  | 104 | negative | previous infection with H1Nx |  |
| Wild boars | 48 | negative | naïve | Surveillance study (39) |
|  | 6 | positive | infected with H5Nx | Wildlife disease monitoring project in Germany 2024/25 (unpublished) |
| Carnivores | 628 | positive / negative | infected with H5Nx / naïve | Wildlife disease monitoring project in Mecklenburg-Western Pomerania, Germany (42) |
| Humans | 8 | positive | vaccinated with H5-specific vaccine (antigens based on clades 1 and 2.1) | Provided by the EMC, Rotterdam, The Netherlands as described in Materials and Methods. |
|  | 20 | negative | naïve | Provided by the Faculty of Medicine and University Hospital Cologne as described in Materials and Methods (40) |
|  | 11 | positive | vaccinated with H5-specific vaccine (antigen based on clade 2.3.4.4b) | Provided by the Faculty of Medicine and University Hospital Cologne as described in Materials and Methods |

**SUPPLEMENTARY TABLE 1:**

Detailed description of samples used for the evaluation of the H5-Nanoluc ELISA. Samples that were included in ROC analysis are indicated in blue.

|  | **H5-Nano** | **ID Screen H5** | **SNT** | **ID Screen NP** |
| --- | --- | --- | --- | --- |
| #1 | positive | negative | positive | negative |
| #2 | positive | negative | positive | negative |
| #3 | positive | negative | positive | negative |
| #4 | positive | negative | negative | negative |
| #5 | positive | negative | positive | negative |
| #6 | positive | negative | negative | negative |
| #7 | positive | negative | negative | negative |
| #8 | positive | negative | positive | negative |
| #9 | positive | negative | negative | negative |
| #10 | positive | negative | positive | positive |
| #11 | positive | negative | positive | negative |
| #12 | positive | negative | positive | negative |
| #13 | positive | negative | negative | negative |
| #14 | positive | negative | negative | positive |
| #15 | positive | negative | positive | negative |
| #16 | positive | doubtful | positive | negative |
| #17 | negative | positive | positive | positive |
| #18 | negative | positive | positive | negative |
| #19 | negative | positive | negative | positive |
| #20 | negative | positive | positive | doubtful |
| #21 | negative | positive | positive | positive |
| #22 | negative | positive | positive | positive |
| #23 | negative | positive | positive | doubtful |
| #24 | negative | positive | negative | positive |
| #25 | negative | positive | positive | positive |
| #26 | negative | positive | negative | doubtful |
| #27 | negative | positive | positive | doubtful |
| #28 | negative | positive | negative | positive |
| #29 | negative | positive | positive | positive |
| #30 | negative | positive | negative | negative |
| #31 | negative | positive | positive | positive |
| #32 | negative | doubtful | positive | negative |
| #33 | negative | negative | positive | negative |

**SUPPLEMENTARY TABLE 2:**

Samples from wild terrestrial carnivores comparatively analyzed with the H5-Nanoluc ELISA, the commercial ID Screen H5- and NP-specific ELISAs and by SNT. Each row shows the results of each test for one sample.
